# Energy restriction in renal protection

**DOI:** 10.1017/S0007114518002684

**Published:** 2018-11-28

**Authors:** Si-Yang Wang, Guang-Yan Cai, Xiang-Mei Chen

**Affiliations:** State Key Laboratory of Kidney Diseases, Department of Nephrology, National Clinical Research Center for Kidney Diseases, Chinese PLA Institute of Nephrology, Chinese PLA General Hospital, Beijing 100853, People’s Republic of China

**Keywords:** Energy restriction, Kidney injury, Ageing, Inflammation, Oxidative stress, Sirtuin type 1: Autophagy

## Abstract

Energy restriction (ER) has been widely studied as a novel intervention, and its ability to prolong life has been fully demonstrated. For example, ER can significantly extend the lifespans of model flies, worms, rodents and other mammals. The role of ER in renal protection has also been elucidated. In preclinical studies, adjusting total energy intake or consumption of specific nutrients has prophylactic or therapeutic effects on ageing-related kidney disease and acute and chronic kidney injury. Amino acid restriction has gradually attracted attention. ER mimetics have also been studied in depth. The protective mechanisms of ER and ER mimetics for renal injury include increasing AMP-activated protein kinase and sirtuin type 1 (Sirt1) levels and autophagy and reducing mammalian target of rapamycin, inflammation and oxidative stress. However, the renal protective effect of ER has mostly been investigated in rodent models, and the role of ER in patients cannot be determined due to the lack of large randomised controlled trials. To protect the kidney, the mechanism of ER must be thoroughly researched, and more accurate diet or drug interventions need to be identified.

In 1935, McCay *et al*.^(^ ^1^ ^)^ discovered for the first time that energy restriction (ER) was able to extend the mean and maximal lifespans of rodents. Recently, in a 20-year study of primates, Colman found that ER beneficially aided resistance to age-related diseases and death^(^ ^2^ ^)^. ER can ameliorate the effects of senescence and age-related diseases without causing malnutrition in many animal models, including invertebrates, rodents and primates. At the same time, reducing intake of specific nutrients rather than overall energy content can also play a key role in these observed effects. Restricting protein and specific amino acids largely contributes to the effects of ER. In addition, the use of energy restriction mimetics (ERM) has gradually gained attention.

Acute kidney injury (AKI) is a common disorder with a high risk of mortality and development of chronic kidney disease (CKD). In 2013, the International Society of Nephrology launched a global target of ‘0by25’ – no patient deaths due to untreated acute kidney failure by 2025 – to improve the diagnosis and treatment of AKI globally^(^ ^3^ ^)^. A systematic review (2004–2012) of large cohort studies published in 2013 on the worldwide AKI prevalence reported that one in five adults and one in three children experienced AKI during a hospital episode of care^(^ ^4^ ^)^. Moreover, a recent large, prospective, multinational study of AKI epidemiology in children and young adults in intensive care units showed that AKI occurred in one-quarter of patients during the first 7 d after intensive care unit admission^(^ ^5^ ^)^. These analyses have raised awareness of AKI among the public, government officials and health care professionals. If effective treatment is not provided promptly, AKI will eventually lead to death or CKD. Age is also an important risk factor for kidney injury. Therefore, finding a way to delay ageing of the kidney and to protect the kidney may have great significance for reducing the occurrence of kidney injury.

Nadon’s study found that ER can prevent or delay the occurrence of age-related nephropathy^(^ ^6^ ^)^. ER also reduces mesangial cell proliferation, stromal hyperplasia and proteinuria and prevent age-related glomerulosclerosis in aged rats^(^ ^7^ ^)^. The same preventive or protective effects against acute and chronic kidney injury are achieved by adjusting total dietary energy content or the ingestion of specific nutritional components. For example, protein restriction (PR) in the diet can improve symptoms of uraemia. In addition, a low-protein diet (plus essential amino acids or keto acids) can improve metabolic disorders in CKD and rectify metabolic acidosis, Ca and P metabolism and other abnormalities, thereby delaying the time of renal replacement therapy; this diet is therefore an important intervention for the treatment of CKD. However, the protective effect and mechanism of ER in renal injury have not been fully elucidated. In this review, we summarise the current knowledge in this regard.

In some studies, dietary restriction (DR) is equivalent to ER, indicating an overall decrease in food consumption. Most DR studies impose a 20–40 % ER, and the duration of this restriction ranges from a few weeks to an entire lifespan^(^ ^8^ ^)^. After the beneficial effects of ER on the body were confirmed by a large number of studies, questions arose concerning whether the reduced intake of certain nutrients, such as proteins, lipids or carbohydrates, induced by ER played a synergistic role in the body. Although some studies have found that restricting carbohydrates, lipids, minerals and other nutrients does not significantly extend lifespan, inhibit tumour development or impose other effects^(^ ^9^ ^,^ ^10^ ^)^, most PR studies have reported longevity benefits. These findings highlighted the specific importance of PR, even though the effect of PR was less than that of ER^(^ ^11^ ^)^. Next, researchers began to explore whether the restriction of a particular amino acid was responsible for the main benefit of PR^(^ ^12^ ^)^.

## Basic study and mechanisms

**Fig. 1:**
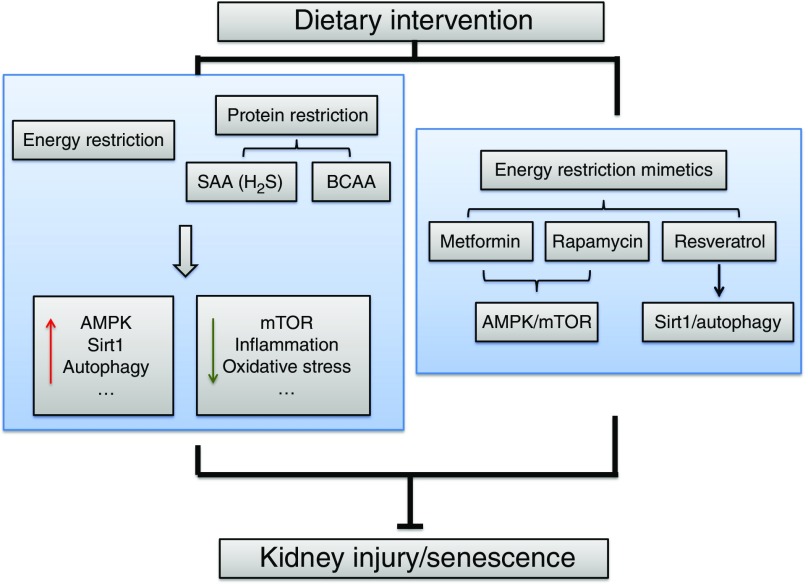
The mechanisms of energy restriction in renal protection. The main identified mechanisms include increased AMP-activated protein kinase (AMPK), sirtuin type 1 (Sirt1) and autophagy and reduced mammalian target of rapamycin (mTOR), inflammation and oxidative stress. SAA, sulphur amino acid; BCAA, branched-chain amino acid.

To observe the effects of ER on the lifespan and kidney disease, studies have focused on basic research with animal models. The identified mechanisms include increased autophagy, reduced inflammation and oxidative stress, improved insulin sensitivity and increased sirtuin type 1 (Sirt1) levels (Fig. 1). The amount (20–40 %) and duration (intermittent or continuous fasting ranging from weeks to the entire lifespan) of ER also vary.

### Ageing-related renal changes

In ER studies, effects on ageing and ageing-related alterations are the primary observational indicators, and these parameters have been used in various animal models, including humans, mice, flies and worms. Currently, most studies on kidney senescence use rodent or primate models. Many studies have shown that ER can delay the ageing-induced incidence of CKD, improve kidney function and delay the incidence of kidney disease. The AMP-activated protein kinase (AMPK) and mammalian target of rapamycin (mTOR) pathways are important for cellular energy metabolism. Increased mTOR expression in the ageing kidneys of rats indicates that mTOR activation can accelerate senescence of the kidney^(^ ^13^ ^)^, whereas AMPK phosphorylation can inhibit this process. Mammalian target of rapamycin complex 1 (mTORC1) inhibition in aged rats can regulate age-related gene changes in the kidney and block age-related chronic progressive nephropathy^(^ ^14^ ^)^. Our previous study also found that stimulation with high glucose induced senescence in rat mesangial cells through AMPK/mTOR pathways^(^ ^15^ ^)^. Our later study showed that short-term ER directly up-regulated AMPK and down-regulated the mTOR signalling pathway, which significantly down-regulated urinary proteins in older rats and delayed tubular epithelial cell senescence and the epithelial to mesenchymal transition (EMT) stimulated by high glucose^(^ ^16^ ^)^. MicroRNA-21 (miR-21) targets the regulation of PPAR*α*, which can promote the EMT in human renal tubular epithelial cells through hypoxia-inducible factor (HIF)-1*α* ^(^ ^17^ ^)^. In rats, long-term ER significantly improves ageing and fibrosis of the kidneys and reduces miR-21 expression in ageing renal tissue or urinary exosomes. A meta-analysis of ER studies revealed that ER delayed the progression of CKD, reduced serum creatinine, urea N and proteinuria levels and improved the survival rate in rodent models by reducing the incidence of diseases, such as CKD^(^ ^8^ ^)^.

Lopez-Dominguez *et al*. studied the effect of fat composition on longevity. The mice were divided into a control group and three 40 % ER groups. The fat sources of the three ER groups were soyabean oil (high in *n*-6 PUFA), fish oil (high in *n*-3 PUFA) or lard (high in SFA and MUFA). This study found that the animals fed lard had increased longevity compared with the mice fed soyabean or fish oil^(^ ^18^ ^)^. This finding suggests that the use of a low proportion of PUFA and a high proportion of MUFA and SFA can maximise the lifespan under ER conditions. Using a ER model, Calvo-Rubio *et al*. studied the contribution of fat sources to changes in the kidney structure. Long-term ER improved the structure of senescent kidneys, as evidenced by the enlargement of the glomerular basal membrane (GBM) and podocyte foot processes (PFP), narrowing of the filtration slits and modification of proximal convoluted tubule cells. In addition, the beneficial effects of ER on GBM thickness, PFP width, autophagy and the mitochondrial mass, size and shape were based on lard as the main fat source^(^ ^19^ ^)^. This result clarifies the critical role of fat sources in delaying kidney ageing.

### Inflammation

During ageing, inflammation and proinflammatory factors gradually increase, which is called the senescence-associated secretory phenotype (SASP). In addition, chronic inflammation is considered a potential risk factor for senescence^(^ ^20^ ^,^ ^21^ ^)^. Inhibiting inflammation may be an important strategy to slow ageing. In mice, chronic inflammation induced by selective knockout of the NF-*κ*B subunit leads to premature ageing via reactive oxygen species (ROS)-mediated exacerbation of telomere dysfunction and cell senescence^(^ ^22^ ^)^. Some studies have revealed that SASP reinforces senescence via increasing ROS in a mechanism similar to interferon-*β* and transforming growth factor-*β* (TGF-*β*)^(^ ^23^ ^,^ ^24^ ^)^. End-stage renal disease (ESRD) patients have an abnormal immune system. Uraemic inflammation is related to mechanisms involved in the ageing process, such as telomere shortening, mitochondrial dysfunction and altered nutrient sensing. These mechanisms may promote a premature ageing process of the uraemic immune system^(^ ^25^ ^)^.

In animals, ER can reduce the level of inflammatory cytokines and mitigate inflammation. The NF-*κ*B transcription and proinflammatory cytokine levels increase with age^(^ ^26^ ^)^. During the renal senescence process, IL-1*β* and IL-18 promote apoptosis in mesangial cells and podocytes, facilitate extracellular matrix deposition and eventually lead to glomerulosclerosis^(^ ^27^ ^)^. A 6-week ER can reduce TNF-*α* expression in the rat liver^(^ ^28^ ^)^. Short-term (4 weeks) ER also reduces expression of a variety of inflammatory factors, improves insulin sensitivity and ultimately relieves renal ischaemia–reperfusion injury. These effects contribute to an increased survival rate^(^ ^29^ ^)^. Pathological studies have also shown that ER can reduce a variety of glomerular and tubular injuries, including proliferation in ageing kidneys, leukocyte infiltration, vascular endothelial thickening, thrombosis, interstitial inflammatory cell infiltration and epithelial cell degeneration, atrophy and interstitial fibrosis. ER blocks the Toll-like receptor 4 (TLR4)/NF-*κ*B signalling pathway by overexpressing the single Ig IL-1-related molecule (SIGIRR), thereby reducing the expression of downstream inflammatory cytokines and slowing inflammation of the kidney^(^ ^30^ ^)^. SIGIRR negatively regulates the TLR4 signalling pathway, inhibits lipopolysaccharide-induced activation of TLR4 and NF-*κ*B and down-regulates expression of leukocyte adhesion molecules, cytokines and angiogenic factors^(^ ^31^ ^)^. SIGIRR is an important negative regulator of inflammation^(^ ^32^ ^)^ that can inhibit renal fibrosis^(^ ^33^ ^)^. The specific mechanism of ER and inflammation in the ageing kidney has yet to be fully elucidated; therefore, SIGIRR may be an important component in the ER-induced anti-inflammatory anti-ageing process and may serve as a new target for slowing ageing.

### Oxidative stress

ROS and other active molecules are produced in excess when the body continues to encounter stress factors. The oxidative and antioxidative systems become unbalanced. This imbalance can be further aggravated if the body presents with additional defects in the antioxidant capacity, eventually leading to injury^(^ ^34^ ^,^ ^35^ ^)^. Therefore, to delay kidney ageing and prevent and treat ageing-related kidney disease, the level of oxidative stress in the body must be reduced, and the redox balance should be maintained.

d-*β*-Hydroxybutyrate (*β*OHB) is an endogenous specific inhibitor of class I histone deacetylases. In addition to administration of exogenous *β*OHB, fasting and ER can increase the production of endogenous *β*OHB, resulting in histone acetylation in mouse tissues and transcription of antioxidant stress factors, such as Forkhead box O3 (Foxo3a) and metallothionein-2^(^ ^36^ ^)^. The most recent literature shows that ER suppresses mitochondrial oxidative stress by up-regulating H_2_S production, which protects the liver and kidneys from ischaemia–reperfusion injury. Further *in vivo* and *in vitro* experiments have shown that restricting the intake of sulphur amino acids (SAA), such as cysteine and methionine, can also mitigate ischaemia–reperfusion injury in the liver and kidney via endogenous H_2_S levels^(^ ^37^ ^)^. SAA restriction reduces mitochondrial oxidative stress in the brain, heart, liver and kidneys^(^ ^38^ ^–^ ^40^ ^)^. Our previous studies discovered that the production of endogenous H_2_S was up-regulated after exposure of ageing male F344 rats to 30 % ER, which resulted in decreased ROS levels, protein carbonylation and malondialdehyde production; in addition, the redox balance was maintained, and kidney ageing was delayed. Furthermore, different durations of ER had different protective effects against oxidative stress in ageing kidneys. Compared with short-term (6 weeks) ER, ER lasting 6 months or the entire lifespan significantly prolonged the lifespan and reduced oxidative stress^(^ ^41^ ^)^.

### Sirtuin type 1 and autophagy

Initially, Sirt1 was identified as a NAD-dependent histone deacetylase^(^ ^42^ ^)^. Subsequent studies have shown that under fasting conditions, Sirt1 regulates energy metabolism and the stress response via acetylation of many internal nuclear transcription factors^(^ ^43^ ^)^. In addition, some studies have demonstrated an effect of ER and Sirt1 on kidney injury and glomerular diseases in ageing models. Research on the protective mechanism of Sirt1 in the kidney under ER conditions is gradually expanding.

Sirt1 is mainly expressed in renal tubules. In mice, Sirt1 activity gradually decreases with age in the kidney and in a mouse model of diabetic nephropathy (DN), but this effect can be reversed by ER^(^ ^44^ ^)^. Compared with the levels in younger animals, 24-month-old mice presented with decreased Sirt1 expression, increased mitochondrial oxidative stress and abnormal mitochondrial morphology in the kidney. Beginning a 40 % ER intervention at 12 months of age can relieve the ageing-associated changes observed in mice^(^ ^45^ ^)^. Long-term ER improves autophagic activity, which also occurs in ageing kidneys. Autophagy provides nutrients and energy by degrading unessential contents within the autophagosome. This process can also prevent cell damage and cell apoptosis by degrading potential toxic proteins^(^ ^46^ ^)^. Autophagy activation has been found to be essential for ER-mediated life extension and anti-ageing effects in both lower animal species and mammals. Short-term ER activates autophagy in renal tubular epithelial cells^(^ ^45^ ^)^. In addition, long-term ER in ageing rats significantly improves autophagy and reduces DNA damage and ageing markers^(^ ^47^ ^)^. Sirt1-mediated autophagy may be critical for the mechanisms of ER. BCL2/adenovirus E1B 19 kDa protein-interacting protein 3 (*Bnip3*) is a gene that activates autophagy. In Sirt1^+/−^ kidneys, autophagic activity is low, and Bnip3 expression is decreased; however, ER activates Sirt1 and FOXO3a transcription and Bnip3-mediated autophagy. The sequestosome 1 (p62/Sqstm1) pathway is involved in autophagy; ER increases p62/Sqstm1 signalling, improves the function of the autophagy system and normalises mitochondrial morphology in addition to rescuing Sirt1 expression^(^ ^44^ ^)^.

In a kidney disease model, Sirt1 activation attenuates cisplatin-induced renal tubular injury. The molecular mechanisms involve inactivating core histone transcription and repairing DNA damage^(^ ^48^ ^)^. An 8-week ER increases Sirt1 expression in the kidneys of aged rats, inhibits renal tubular epithelial cell apoptosis and reduces cisplatin-induced AKI^(^ ^49^ ^)^. Microinflammation has recently been implicated in the pathogenesis of or treatment for DN. Sirt1 also has anti-inflammatory effects in many organs^(^ ^50^ ^)^, suggesting that Sirt1 is involved in ER-related anti-inflammatory molecular mechanisms. Moreover, Sirt1 up-regulates cyclo-oxygenase-2 expression in the kidney by interfering with HIF-2*α* activation, increases peroxidase activity and inhibits the expression of TGF-*β*-Smad3 (mothers against decapentaplegic homolog 3), NF-*κ*B, p53 and other pathways to prevent inflammation, apoptosis, fibrosis and proliferation in the kidney^(^ ^51^ ^)^.

## Protein restriction

A large number of studies have proven the protective effect of ER, but it remains unclear whether a specific nutrient in the diet mediates the main effect of ER. After comparing multiple nutrients, PR has emerged as potentially important for ER^(^ ^11^ ^)^.

Studies have shown that PR can achieve effects similar to those of ER. FOXO3, hepatocyte nuclear factor 4 and high mobility group A1 expression in the kidneys of 3-month-old male C57 mice was increased by 30 % ER and 3-d PR, thereby reducing ischaemia–reperfusion injury in the kidney^(^ ^52^ ^)^. In Dahl SS rats, a high-protein diet exacerbates the deterioration in blood pressure that accompanies extensive glomerular inflammatory cell infiltration^(^ ^53^ ^)^. PR also has an effect on the mTOR pathway that is similar to that of ER. In male mice, PR and ER both contribute to the benefits of short-term ER against renal ischaemic–reperfusion injury in a manner that is partially dependent on AMPK activation and mTORC1 repression^(^ ^54^ ^)^. As a nutrient-associated pathway, AMPK/mTOR plays a key role in the protective effect in the kidney during PR or ER^(^ ^54^ ^)^, and mTOR has been shown to be a central regulator of cell growth. mTOR activation plays a pivotal role in DN and the development of ESRD^(^ ^55^ ^)^. Thus, inhibition of mTOR may be a strong potential therapeutic target for acute and chronic kidney injury.

Regarding specific amino acids, methionine restriction achieves effects similar to those of PR, such as reducing mitochondrial ROS production, free radical leakage and the mitochondrial DNA 8-oxo-2′-deoxyguanosine levels^(^ ^11^ ^)^. Chronic or excessive methionine supplementation also increases renal tubulointerstitial damage^(^ ^56^ ^)^. In the rat liver and kidney, methionine supplementation increases the two methionine-derived circulating metabolites (*S*-adenosylmethionine (SAM) and S-adenosine homocysteine (SAH)) and induces some damage, such as the generation of mitochondrial free radicals and oxidative DNA damage. Mitochondrial and DNA damage are related to overproduction of SAM and SAH^(^ ^57^ ^)^. A 40 % methionine restriction reduces mitochondrial ROS production, free radical release, renal mitochondrial DNA oxidative damage and mitochondrial protein oxidation-specific markers (i.e. lipoxidation and glycoxidation). This methionine restriction also reduces the expression levels of mitochondrial respiratory chain complexes I, III and IV, apoptosis-inducing factor and renal mitochondrial complex IV in the brain^(^ ^58^ ^,^ ^59^ ^)^. In 2015, Hine *et al*. found that ER increased production of endogenous H_2_S in the liver and kidney through increased synthase cystathionine-*γ*-lyase (CGL) and cystathionine-*β*-synthase expression, which reduced ischaemia–reperfusion injury by restoring the redox balance. Methionine and cysteine supplementation abrogated H_2_S production and the significant benefits of combined protein/energy restriction^(^ ^37^ ^)^. This finding provides a new mechanism for the protective effect of methionine restriction. Although toxic at high levels, H_2_S is produced at low concentrations due to cysteine or homocysteine degradation by CGL and has a beneficial effect on the vasculature and brain. H_2_S acts as a signalling molecule to reduce blood pressure^(^ ^60^ ^)^ and prevent neurodegeneration^(^ ^61^ ^)^. Exogenous H_2_S can also extend the lifespan of worms^(^ ^62^ ^)^ and induce suspended animation-like states in mammals^(^ ^63^ ^)^. The increase in H_2_S production induced by SAA restriction can activate the proangiogenic pathway through general control non-derepressible 2/activating transcription factor 4 (GCN2-/ATF4) in endothelial cells, which increases capillary density in mouse skeletal muscle^(^ ^64^ ^)^. In addition, an interaction occurs between H_2_S and Sirt1 during regulation of angiogenesis in old mice to reverse age-associated loss in muscle vascular density. H_2_S has been hypothesised to delay ageing in part by activating Sirt1, which is a major lifespan regulator^(^ ^65^ ^)^. As the only source of endogenous H_2_S, methionine restriction may have a good application prospect for renal protection.

Leucine restriction (LR) for 7 d resulted in body weight loss and decreased adiposity^(^ ^66^ ^)^. A specific reduction in the consumption of three branched-chain amino acids (BCAA; leucine, isoleucine and valine) by mice can improve metabolic health^(^ ^67^ ^)^. LR can replicate some, but not all of the effects of methionine restriction, such as decreasing body and fat mass, elevating lipid cycling in white adipose tissue and improving whole-body glucose metabolism and hepatic insulin sensitivity^(^ ^68^ ^)^. However, changes in the kidney after LR have not been investigated. Insulin resistance affects many aspects of kidney function, including renal haemodynamics, podocyte viability and tubular function^(^ ^69^ ^)^. Thus, BCAA restriction may be a promising treatment for DN in addition to LR.

In CKD, PR can reduce proteinuria and decrease urea N levels and changes in the kidney; however, excessive PR decreases the serum albumin level and slows growth. Adding keto acids can correct these results and have better effects than single PR^(^ ^70^ ^)^. Kruppel-like factor 15 (KLF15) is a transcription factor that is reduced in heart fibrosis. KLF15 was also decreased in a CKD model. PR increases the KLF15 level in healthy kidneys and partially recovers the KLF15 level in kidneys with residual renal function^(^ ^71^ ^)^. This finding demonstrates the potential role of PR in anti-renal fibrosis.

## Energy restriction mimetics

### Metformin

ERM are drugs that increase the consumption of food and water without adjusting energy intake and are believed to achieve the same effects as ER, such as prolonging the lifespan and reducing age-related diseases^(^ ^72^ ^)^. Metformin is a representative ERM drug.

Derivatives of biguanide compounds, such as buformin, metformin and phenformin, improve the stability of blood glucose and demonstrate preventive effects on ageing. These drugs can also induce metabolic effects by activating AMPK and inhibiting mTOR^(^ ^73^ ^)^. Metformin slows ageing and extends the lifespan of female spontaneously hypertensive rat mice^(^ ^74^ ^)^. In addition, metformin impedes the TGF-*β*-induced loss of the epithelial marker E-cadherin in MCF-7 breast cancer cells and prevents the accumulation of the mesenchymal marker vimentin in Madin–Darby canine kidney (MDCK) cells^(^ ^75^ ^)^. In Zucker diabetic fatty rats, metformin decreases oxygen consumption and the ATP levels, increases the intracellular oxygen partial pressure and reduces HIF-1 expression, pimonidazole staining and renal damage^(^ ^76^ ^)^. In our experiments, metformin inhibited the expression of HIF-1*α* and its target genes in human renal proximal tubular epithelial cells^(^ ^16^ ^)^. Studies on the renal-protective effects of metformin have mainly been conducted with diabetic patients. A recent systematic review showed that metformin reduced the all-cause death rate in diabetic patients with CKD (glomerular filtration rate (GFR) <60 ml/min per 1·73 m^2^)^(^ ^77^ ^)^. However, the application of this result in patients with DN is limited, since they are at risk for lactic acidosis. Because the clinical application of metformin is still mainly focused on the treatment of diabetes mellitus, the effects of metformin on other diseases, such as cancer and lifespan, remain controversial. The US National Institute on Aging Interventions Testing Program^(^ ^78^ ^)^ evaluated the influence of different agents on longevity in genetically heterogeneous mice. When used alone, metformin showed a non-significant effect on the median lifespan, and the site-specific effects were indistinguishable from chance, which was different from some past reports. This discrepancy was likely due to the use of genetically heterogeneous mice, the initiation time of metformin treatment, evaluation of male and female mice, analysis at three independent sites and the choice of statistical method. Interestingly, metformin combined with rapamycin robustly extended the lifespan, which was an added benefit, compared to rapamycin alone, indicating that metformin might have some positive effects on the lifespan. However, large-scale clinical trials are needed to evaluate the effects on longevity and renal disease patients.

### Rapamycin

Rapamycin is an Food and Drug Administration-approved drug that is used to coat coronary stents and prevent organ transplant rejection, such as kidney and islets^(^ ^79^ ^)^. Rapamycin specifically inhibits mTOR and leads to very significant inhibition of renal cyst growth as a ERM for renal protection. Autophagy is regulated by mTORC1 and other nutrient-responsive intracellular signals; rapamycin, which improves glomerular lesions in experimental DN, can activate autophagy^(^ ^80^ ^)^. Autosomal-dominant polycystic kidney disease (ADPKD) is a common cause of ESRD. The mTOR pathway has been shown to be activated in ADPKD, and inhibition of mTOR with rapamycin decreases the development of ADPKD in mice^(^ ^81^ ^,^ ^82^ ^)^. In diabetic obese db/db mice, which are characterised by mTOR activation, systemic administration of rapamycin markedly ameliorates pathological changes and renal dysfunctions^(^ ^55^ ^)^. Inhibition of the mTOR pathway with rapamycin and activation of the AMPK pathway with metformin also lead to decreased cystogenesis in MDCK cell and metanephric cultures^(^ ^83^ ^)^. Due to its outstanding effect on mTOR inhibition, rapamycin may have a good application prospect for kidney disease, especially DN and ADPKD.

### Resveratrol

Resveratrol, which is a monomer of polyphenol found in grapes, can also simulate the biological effects produced by ER, such as reducing oxidative stress in renal injury. Recent studies by Kitada have shown that resveratrol can inhibit Smad3 acetylation by activating Sirt1 in unilateral ureteral obstruction (UUO) animal models or cultured cells, leading to a decrease in TGF-*β*1-induced type IV collagen and fibronectin. This effect can alleviate renal injury during hypoxic stress^(^ ^84^ ^)^. Resveratrol can also improve the activity and function of antioxidants by activating Sirt1 during acute and chronic kidney injury^(^ ^85^ ^)^. For AKI models, resveratrol can be used from 10 to 100 mg/kg and the protective effects can be produced within 3–5 d of injection^(^ ^86^ ^–^ ^88^ ^)^. This shows that resveratrol can have the effect on renal protection in a short time. However, the long-term effect of resveratrol on the renal repair function after AKI is relatively limited, and can be one of the research directions in the future. The beneficial effects of ER involve impacts on the function of Sirt1. Sirt1 is a key molecule that regulates renal protective effects in various renal disorder models. The protective effects include maintenance of glomerular barrier function, anti-fibrotic effects, anti-oxidative stress effects and regulation of mitochondrial function and energy metabolism^(^ ^89^ ^)^. Resveratrol is the most potent Sirt1 activator. Sirt1 is highly expressed in the kidney, especially in proximal tubular cells. Moreover, renal proximal tubular cells are susceptible to injury, and senescence may occur first in tubular cells. These facts all make resveratrol a superior ERM for the kidney. In diabetic mouse models, Sirt1 expression is significantly reduced in the proximal tubules of wild-type mice^(^ ^89^ ^)^. Previous studies have clearly demonstrated that resveratrol can improve DN in several animal models of type 1 and 2 diabetes. Resveratrol prevents DN in db/db mice by phosphorylation of AMPK and activation of Sirt1 to prevent mesangial cell apoptosis and oxidative stress in the kidney^(^ ^90^ ^)^. Therefore, resveratrol may have a protective effect on both AKI and CKD.

The effect of resveratrol may be mediated through a Sirt1-independent mechanism. The mTOR signalling pathway is associated with DN and polycystic kidney disease^(^ ^91^ ^,^ ^92^ ^)^. Resveratrol increases the interaction between mTOR and its inhibitor DEP (dishevelled, egl-10, pleckstrin) domain-containing mTOR-interacting protein, resulting in mTOR inhibition^(^ ^93^ ^)^. Therefore, resveratrol may also achieve its renal protective effect through mTOR. Our study also found that resveratrol mitigated high glucose-induced human proximal tubular cell senescence *in vitro* and prevented the formation of paracrine vesicles containing miR-21, which promoted the EMT process^(^ ^16^ ^)^.

## Clinical studies

### Energy content and proteins

Clinical studies on ER have focused on metabolism and insulin sensitivity as well as oxidative stress, inflammation, cardiovascular risk factors and corresponding indicators of patients with obesity or diabetes. Clinical studies on the renal-protective effects are more concentrated on PR in CKD patients and on ER in DN patients.

Clinical studies on ER are limited, and thus the role of ER in CKD patients cannot be determined due to the lack of large randomised controlled trials (RCT). Giordani *et al*.^(^ ^94^ ^)^ analysed the glomerular filtration rate (GFR) of fourteen obese patients with type 2 diabetes and stage 2 CKD; the study concluded that 7 d of a low-energy diet significantly improved the GFR of these patients. Therefore, short-term ER may improve renal function. In addition, ER for 12 weeks decreased body weight and the serum creatinine and cystatin C levels in obese patients with DN and increased the GFR^(^ ^95^ ^)^. In 2003, Morales *et al*.^(^ ^96^ ^)^ studied the weights and proteinuria of CKD patients. A total of thirty overweight or obese patients with albuminuria, including DN patients and patients with other manifestations of proteinuria, were randomly divided into two groups. The patients were given either a normal diet or a low-energy diet. After 5 months, the urine protein was decreased by 30 % in the ER group (from an average of 2·8–1·9 g/d). The renal function remained stable in the ER group but had a worsening trend in the free diet group; however, the renal function was not significantly different between the two groups.

Ageing progresses so slowly that clinical dietary interventions on ageing take a very long time and may be difficult to apply in people due to inconvenience issues. In addition, ER may still cause some negative effects, such as a decline in the quality of life, and people may not adhere to long-term ER. Although ER has proven to be beneficial for longevity in animal models ranging from worms to primates, long-term ER is difficult to achieve in people. For people, ERM may be more practical than ER^(^ ^97^ ^)^.

The PR program for kidney disease may have been performed for the first time in patients by Kempner^(^ ^98^ ^)^. Kempner thought that the kidney played a key role in malignant hypertension. He began to treat patients with malignant hypertension with a diet composed of nothing but rice and fruit. Most patients were able to reduce their blood pressure. However, he also found that the treatment of kidney disease and hypertensive with the rice diet was either ineffective or dangerous unless it was conducted under rigidly controlled conditions^(^ ^99^ ^)^. Therefore, the time and level of PR are essential for the regimen for renal disease patients. PR reduces uraemic toxins, blood urea N levels, the acid load, oxidative stress and the phosphate load and also improves insulin resistance and control over metabolic bone disease and ameliorates anaemia^(^ ^100^ ^–^ ^103^ ^)^. Depending on the protein source, PR may delay progression of CKD mainly by reducing the Na, K and P levels. Adding keto acids to PR during CKD stages 4–5 or before or during dialysis aids in the avoidance of malnutrition and improves the patient prognosis. Although this method improves endothelial function, no obvious reduction in cardiovascular risk has been found^(^ ^70^ ^)^. PR did not significantly decrease blood P levels, and a vegetarian diet only reduced blood P by 3 mg/l. The modification of diet in renal disease study also showed that the risk of death for patients with advanced CKD receiving a very low-protein diet of 0·58 g/kg body weight per d was higher than that for patients receiving a low-protein diet of 0·6–0·8 g/kg per d. The clinical research results related to PR are inconsistent. Some studies indicate that changes in protein intake will not cause significant changes in renal function, disease progression or the risk of dialysis and death^(^ ^104^ ^–^ ^106^ ^)^. However, most studies show that PR delays the decline in renal function and improves the renal survival rate. Although the results vary, they still support dietary intervention for patients with early CKD and DN.

### Salt

Urinary albumin excretion is an important independent risk factor for the development and progression of kidney disease as well as for CVD, such as diabetes and hypertension. A number of RCT studies have shown that limiting salt intake significantly reduces urinary albumin and the albumin:creatinine ratio (9·1 mg/24 h and 0·66 mg/mmol, respectively) and improves arterial compliance in different ethnic patients with mild hypertension^(^ ^107^ ^)^. In CKD patients with proteinuria, salt restriction reduces the vascular endothelial growth factor C and N terminal pro B type natriuretic peptide levels, impairs increases in the extracellular volume and decreases hypertension^(^ ^108^ ^)^. In patients with high blood pressure, urine protein can be reduced from 93 (SD 48) to 75 (SD 30) mg/24 h^(^ ^109^ ^)^. However, endpoint events were not observed. In 2015, McMahon *et al*.^(^ ^110^ ^)^ conducted a systematic analysis of eight previous studies and found a lack of evidence supporting the long-term effects of low salt intake in CKD patients and the direct impact of this modification on end points such as mortality and ESRD. A decrease in blood pressure and proteinuria was identified, which was beneficial for reducing the incidence of ESRD and cardiovascular events in CKD patients.

### Phosphorus

Hyperphosphataemia accelerates renal failure. Moreover, accelerated ageing is associated with high serum inorganic phosphate (Pi) levels. The Psychological, Social and Biological Determinants of Ill Health pSoBid cohort study performed by the University of Glasgow explored the impact of dietary P intake on human age-related health. The data indicate that high Pi levels are associated with features of biological ageing, such as decreasing telomere lengths and increases in inflammation and DNA hypomethylation. For renal health, Pi levels have a close relationship with renal function based on estimated glomerular filtration rate (eGFR) measurement. Some subjects with high Pi levels have eGFR values indicative of incipient or early onset CKD^(^ ^111^ ^)^. Pi overload may lead to tubular injury and interstitial fibrosis^(^ ^112^ ^)^. Serum phosphate and its product, calcium phosphate, were increased significantly in uraemic rats fed high P for 6 months, resulting in a mortality rate of 71·4 %. In rats fed a low-P diet, the parathyroid hormone and fibroblast growth factor-23 (FGF-23) levels were significantly decreased in the blood, and the mortality rate was only 5·9 %. In addition to reducing vascular calcification, a low-P diet can relieve renal interstitial fibrosis, glomerular sclerosis and inflammatory cell infiltration^(^ ^113^ ^)^. High levels of hyperphosphataemia are associated with CVD events in CKD and ESRD populations as well as in healthy people. In dialysis patients, high P and death are positively correlated. FGF-23 is the most important P-regulating hormone and increases P excretion when the serum phosphate level is too high, but FGF-23 also decreases the vitamin D levels, promotes ventricular hypertrophy, accelerates the deterioration of renal function and increases cardiovascular mortality.

Dietary P includes both organic and inorganic P. Organic P comes from protein sources, whereas inorganic P originates from additives or preservatives. Although the body absorbs approximately 90 % of inorganic P, the absorption of organic P is only 40–60 %^(^ ^114^ ^)^. In an effort to limit P, PR may increase the incidence of mortality and malnutrition in CKD and dialysis patients. The Kidney Disease Outcomes Quality Initiative recommends a P intake of 800–1000 mg/d for CKD patients^(^ ^115^ ^)^. Selamet *et al*.^(^ ^116^ ^)^ found that phosphates in food were not associated with the incidence of ESRD or CVD or with non-CVD or all-cause mortality in patients with stage 3–5 CKD; in addition, serum P levels and health benefits were not related to P restriction. The National Health and Nutrition Examination Survey III showed that the serum P concentration increased by only 0·06 mg/l per 500 mg/d intake^(^ ^117^ ^)^. *JAMA* published a study in which haemodialysis patients were educated about the products that contained high inorganic P (i.e. soda water), and the intake of these products was limited. This approach significantly reduced the serum P level by 10 mg/l^(^ ^118^ ^)^. Therefore, to avoid the adverse events caused by low protein intake, reasonable P restrictions should mainly focus on inorganic P. In addition, P binders can be used to emulate P restriction in patients with advanced CKD^(^ ^114^ ^)^.

## Summary

ER protects the kidney in different ways, and ERM are gradually being studied in more depth. Most of the studies in this review focus on rodent model. There are considerable differences between rodents and humans with respect to genetics, anatomy, physiology and metabolism, as well as the structure of the kidney. For example, there is a multilobular, multipapillary architecture in the kidneys of humans and mini pigs, whereas mice, rats, dogs and rabbits have unilobular, unipapillary kidneys^(^ ^119^ ^)^. Therefore, the rodent model results cannot be completely equivalent to human research. However, the protective effect of ER on kidney has been studied in humans, and some similar results can be reached. Therefore, most of the studies about rodent model are also useful for clinical research. As most studies on ER have employed basic research, translating the results to clinical practice is difficult. Clinical trials require volunteer enrolment, and the results are closely associated with the subjects’ compliance. Achieving the desired clinical results with mandatory ER may be difficult. In addition, the best ER application, such as the optimal duration and starting time, ER personalisation programs for unique groups of people and patients, the choice of either a general ER or restriction of a specific nutrient, the ethical issues involved and the overall impact on the patient’s situation, must be discussed. The lack of prospective clinical trials and the suitability of ERM for clinical use are worthwhile issues for continued discussion. In the future, we hope to propose a more accurate diet or drug intervention program through in-depth studies on the mechanism of DR.
